# A prospective study on the evaluation of a novel immunochromatographic card for the rapid detection of five carbapenemase enzymes in metallo-beta-lactamase producers

**DOI:** 10.1099/acmi.0.001024.v3

**Published:** 2025-09-10

**Authors:** Jyotsna Agarwal, Vikramjeet Singh, Avneesh Sharma, Manodeep Sen, Anupam Das

**Affiliations:** 1 Department of Clinical Microbiology, MD Microbiology, Dr Ram Manohar Lohia Institute of Medical Sciences, Lucknow, Uttar Pradesh, India; 2 Department of Clinical Microbiology, MD Microbiology, Sanjay Gandhi Postgraduate Institute of Medical Sciences, Lucknow, Uttar Pradesh, India

**Keywords:** Advanced Expert System, IMP, KPC, metallo-beta-lactamase, NDM, novel immunochromatography card, O.K.N.V.I. card, OXA, PCR, VIM

## Abstract

**Objective.** To evaluate the performance of a novel immunochromatographic (IC) card test (TRURAPID^®^ O.K.N.V.I. RESIST-5) for rapid detection of five carbapenemase enzymes in metallo-beta-lactamase (MBL)-producing organisms, compared to real-time PCR and an Advanced Expert System (AES).

**Methods.** Clinically isolated 100 non-duplicates of multidrug-resistant Gram-negative bacilli expressing MBL production were tested using the novel IC card, real-time PCR and the Vitek-2 AES. Sensitivity, specificity and turnaround time were evaluated.

**Results.** The novel IC card showed high sensitivity for detecting NDM (93%) and KPC (91.7%) carbapenemases, but lower sensitivity for OXA-48 (60%), VIM (67%) and IMP (33%) compared to PCR. It had a rapid turnaround time of 15–20 min versus 5–7 h for PCR and 18–22 h for AES.

**Conclusion.** The novel IC card offers a rapid, cost-effective approach for detecting carbapenemases, particularly NDM and KPC, in clinical microbiology practice. It may be beneficial in resource-limited settings where these enzymes are prevalent. Considering the limited sensitivity for the IMP and VIM genes, this warrants confirmatory testing by PCR. Further evaluation is needed to assess its role as a screening or confirmatory test, especially during nosocomial outbreaks.

**Impact Statement**

This article evaluates a novel immunochromatographic (IC) card test for rapid detection of five carbapenemase enzymes in metallo-beta-lactamase-producing bacteria. The novel IC test showed high sensitivity for NDM, OXA and KPC enzymes, which is pertinent in the context to the Indian subcontinent. It offers a rapid 15–20 min turnaround time versus hours for other methods, potentially beneficial in resource-limited settings in a fight against carbapenem resistance.

## Data Summary

No sequence is generated in this data. No code or software is generated.

## Introduction

The worldwide spread of micro-organisms resistant to carbapenems in hospital settings is a grave threat to human health, as this leaves clinicians with very limited therapeutic options [1,2]. The main mechanism for carbapenem resistance is the production of carbapenemase enzymes, which are frequently horizontally transferred among bacterial species. These carbapenemases and their variants reside on transposons, plasmids and pathogenicity islands known as mobile genetic elements [3]. Thus, carbapenemase-producing (CP) organisms, principally *Enterobacterales*, as well as glucose non-fermenting bacilli, are becoming more common worldwide, especially in India [4,7]. Identifying and characterizing carbapenemases using broth and agar dilution methods employing resistant phenotypes and molecular methods by utilizing PCR could prove difficult, time-consuming and expensive [8,10]. TRURAPID^®^ O.K.N.V.I. RESIST-5 Rapid test (3B Blackbio Dx Ltd, India) is a new and efficient approach to identifying five targeted carbapenemase genes in 15 min using colloidal gold nanoparticles (NPs) and membrane technology. The current research evaluates this unique immunochromatography card test by employing real-time PCR and Advanced Expert System (AES; Vitek-2, bioMérieux, France).

## Methods

### Study design

Hospital laboratory-based prospective research was conducted from November 2023 to May 2024. The study was duly approved by the Institutional Ethics Committee (IEC No. 173/22; 27 Feb 2023). During the study period, various samples from ICU patients received in the microbiology department included catheter urine, blood culture samples, bronchoalveolar lavage, pus and other body fluids. All samples except urine were inoculated on blood agar and MacConkey agar, while urine was inoculated on chrome agar. Positive-flagged blood culture bottles were inoculated on blood and MacConkey agar. The plates were then incubated at 37℃ for 24 h, and isolated Gram-negative bacilli (GNB) were then processed for study. The analysis included 100 non-duplicate randomly selected clinical isolates of multidrug-resistant GNB phenotypically expressing metallo-beta-lactamase (MBL) by Imipenem-EDTA combined disc method from ICU patients. Bacterial isolates included both the *Enterobacterales* and non-fermenting Gram-negative bacteria.

### Bacterial DNA extraction

The overnight bacterial culture was used for DNA extraction using HiMedia^®^ HiPurA Bacterial Genomic DNA Purification Kit. It was performed based on spin column method centrifugation in accordance with the manufacturer’s instructions. Bacterial DNA was isolated and eluted from the columns in 200 µl elution buffer and stored in a mini Eppendorf tube at −20 °C until further use.

### Bacterial identification and MDR MBL screening

Bacterial identification was done using MALDI-TOF MS (bioMérieux, France). Kirby–Bauer disc diffusion and automated antimicrobial susceptibility testing AES (Vitek-2, bioMérieux, France) were used to pick multi drug resistant (MDR) isolates. Multi-drug-resistant isolate proved resistant to at least one antibiotic in three or more classes. Vitek-2 AES is an artificial intelligence-driven software tool that analyses MIC results of antimicrobials and checks for inconsistencies in antimicrobial susceptibility. It recognizes susceptibility patterns that indicate specific phenotypes and interprets results before releasing reports.

Imipenem-EDTA combined disc synergy test (CDST-Imipenem) has been employed to screen disc diffusion results for MBL production. We utilized Imipenem (10 µg) and Imipenem with EDTA (10 µg+750 mg) discs. Incubated plates at 37 °C for 18–24 h have been classified as MBL-positive if the inhibition zone increased by ≥7 mm with the Imipenem-EDTA disc compared to the Imipenem disc only [11].

### Real-time PCR

The enzyme-based spin column (make name) centrifugation method was employed for extracting bacterial DNA from overnight colonies grown on Mueller–Hinton agar. Once the DNA was extracted, Polymerase chain reaction was performed as per kit manufacturer's recommendation which included Denaturation (95°C for 10 min), followed by 45 cycles (60°C for 60s) for annealing along with amplicons extension then finally cooling (4°C); as per the manufacturer’s recommendations, PCR had been conducted. For identifying specific regions of genes encoding carbapenemase enzymes (NDM, KPC, IMP, OXA-48 and VIM), HiMedia’s Hi-PCR^®^ Carbapenemase Gene (Multiplex) Probe PCR Kit has been utilized in the current investigation along with positive and negative control for each gene provided in the kit. Table 1 demonstrates the Bio-Rad Cfx96 Thermal Cycler test. Results of this PCR were used as a reference gold standard because with each run of PCR, we also put controls to validate the amplification graph.

**Table 1.** Details of primer used for detection of various carbapenemase genes along with amplicon sizes

Gene	Probe	Forward sequence (5′–3**′)**	Reverse sequence (5′–3**′)**	Amplicon size
OXA-48	Cy5.5	TATATTGCATTAAGCAAGG	CACACAAATACGCGCTAACC	848 bp
KPC	FAM	TGTCACTGTATCGCCGTC	GTCAGTGCTCTACAGAAAACC	1,011 bp
NDM	HEX	CACCTCATGTTTGAATTCGCC	CTCTGTCACATCGAAATCGC	984 bp
VIM	Texas Red	GATGGTGTTTGGTCGCAT	CGAATGCGCAGCACCAG	390 bp
IMP	Cy5	GGAATAGAGTGGCTTAAYTCTC	CCAAACYACTASGTTATCT	188 bp

### TRURAPID^®^ O.K.N.V.I. RESIST-5 Rapid test (3B Blackbio Dx Ltd, India)

**Fig. 1.**
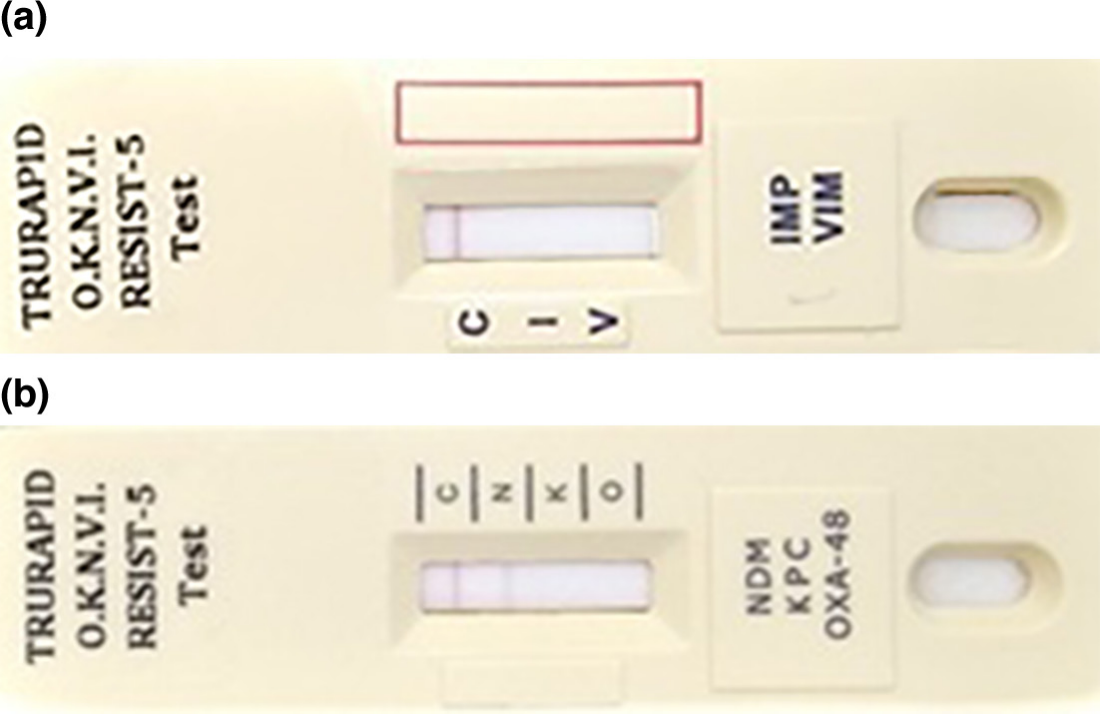
Lateral-flow cassettes are two in number. Each cassette contains one sensitized strip. (a) contains nitrocellulose membrane sensitized with gold NP monoclonal antibody directed against IMP carbapenemase (‘I’ line) and VIM carbapenemase (‘V’ line) and a control capture reagent (‘C’ line). (b) is directed against NDM carbapenemase (‘N’ line), KPC carbapenemases (‘K’ line) and OXA-48-like carbapenemase (‘O’ line) and a control capture reagent (‘C’ line).

This novel immunochromatographic (IC) card assay consists of two lateral-flow cassettes designed to identify five specific carbapenemases (Fig. 1). One cassette detects IMP and VIM, while the other identifies OXA-48-like, KPC and NDM enzymes. This test utilizes membrane technology incorporating various colloidal gold NPs.

Following the manufacturer’s instructions, which are briefly explained here, the TRURAPID^®^ O.K.N.V.I. RESIST-5 assay has been performed: first, we placed 10–12 drops of sample buffer [which is a saline solution with TRIS, NaN3 (<0.1%), detergent and buffered to pH 7.5] in a 2 ml microcentrifuge tube. Next, using a disposable applicator, we picked one to two bacterial colonies from Mueller–Hinton agar (HiMedia, Mumbai, India) and immersed them in the LY-A buffer. Colonies were mixed thoroughly with the applicator. The tube was securely attached to the dropper and vortexed to homogenize the bacterial colony. Each cassette (OXA-48, KPC, NDM and B for IMP and VIM) contained three drops of diluted isolates slowly dispensed into each well. The test reaction was allowed to occur for a maximum of 15 min before reading the results. Results for both cassettes have been determined as follows: a dark brownish line at control (C), rather than other lines, proved negative. At OXA-48-like, KPC, NDM, VIM and/or IMP, a positive test line with a ‘C’ line appeared dark brownish. For the negative control, we used ATCC *Escherichia coli* 25922 strains, and for the positive control, we used known clinical laboratory internal control strains of *E. coli* and *Klebsiella pneumoniae*, for which various carbapenemase genes had already been determined by PCR. Positive and control bands were visually interpreted by two independent observers.

## Results

During the study period, various samples from ICU patients received in the microbiology department included catheter urine, blood culture samples, bronchoalveolar lavage, pus and other body fluids.

### Organism distribution

Among the randomly selected 100 culture-positive MDR MBL isolates, 37 had been *E. coli*, 36 had been *K. pneumoniae*, 11 had been *Pseudomonas aeruginosa*, 10 had been *Acinetobacter baumannii* and 2 each were *Enterobacter* spp., *Citrobacter* spp. and *Proteus* spp. These isolates were randomly selected as per our hospital’s local epidemiology and antibiogram policy.

1. *E. coli* – Among 37 tested isolates, PCR detected 32 OXA-48, 37 NDM and 1 KPC. PCR failed to detect the presence of VIM and IMP in *E. coli* isolates tested. Novel IC card detected 37 OXA-48 and its variant in all isolates, 35 isolates also showed the presence of the NDM gene and 2 isolates each had KPC and VIM; IMP was reported in one isolate. Based on phenotype, AES was able to report 33 producers of OXA-48, 34 of NDM, 1 of KPC and 1 each of VIM- and IMP-producing isolates (Table 2).
2. *K. pneumoniae* – PCR detected the presence of OXA-48, NDM, KPC, VIM and IMP in 31, 36, 3, 1 and 1 in *K. pneumoniae* MBL isolates, respectively. In comparison, novel IC cards detected OXA-48, NDM and KPC in 25, 33 and 4 isolates. The novel IC card failed to detect VIM and IMP production in any of the *Klebsiella* tested.
3. *P. aeruginosa* – PCR showed better performance in *P. aeruginosa* isolates by detecting OXA-48, NDM and KPC isolates in 5, 11 and 1 isolate/s. The novel IC card detected OXA-48, NDM and KPC in 4, 10 and 1 isolate/s.
4. *A. baumannii –* Among ten isolates tested, PCR detected OXA-48, NDM and KPC in eight, ten and two isolates, respectively. However, the novel IC card detected one, ten and four isolates of OXA-48, NDM and KPC, respectively.

### Type of MBL producer detection

PCR could detect multiple genes responsible for MBL production in 81 isolates, and only 19 isolates were found to have a single gene responsible for MBL production (Table 3). Similar results were obtained in AES, where 74 and 26 isolates were observed to have multiple and single genes responsible for MBL production, respectively. Novel IC card O.K.N.V.I. Resist-5 was able to report a single gene responsible for MBL production in 50 isolates and multiple genes responsible for MBL production in 50 isolates.

### Sensitivity and specificity

The positive predictive value (PPV) and the negative predictive value (NPV) of the novel IC card have been shown in Table 4 in comparison to PCR and AES as an expanded diagnostic platform.

### Turnaround time and cost of investigation

TRURAPID^®^ O.K.N.V.I. RESIST-5 assay had an upper hand when it came to turnaround time (TAT) compared to PCR or AES. After the appearance of colonial growth on the agar plate, the time taken for the novel IC card to detect carbapenemase production is 15–20 min, whereas PCR requires 5–7 h (including lysis, extraction and then amplification process). AES takes a maximum TAT of 18–22 h for detecting the MBL phenotype.

**Table 2.** Comparison of various MBL gene detections from PCR, AES and NICC methods

Organism	OXA-48			NDM			KPC			IMP			VIM		
	NICC	PCR	AES	NICC	PCR	AES	NICC	PCR	AES	NICC	PCR	AES	NICC	PCR	AES
*E. coli* (*n*=37)	37	32	33	35	37	34	2	1	1	1	0	1	2	0	1
*Klebsiella* spp. (*n*=36)	25	31	28	33	36	34	4	3	3	0	1	1	0	1	1
*P. aeruginosa* (*n*=11)	4	5	5	10	11	10	1	1	1	0	0	0	0	0	0
*A. baumannii* (*n*=10)	1	8	8	10	10	10	4	2	3	0	0	0	0	0	0
*Citrobacter* spp. (*n*=2)	2	2	2	1	2	2	0	1	1	0	1	1	0	0	0
*Enterobacter* spp. (*n*=2)	0	1	1	2	2	2	0	0	0	0	0	0	0	0	0
Tribe Proteae (*n*=2)	1	2	1	2	2	2	0	0	0	0	0	0	0	0	0

AES, Advanced Expert System Vitek-2; IMP, imipenemase; KPC, *Klebsiella pneumoniae* carbapenemase; NDM, New Delhi metallo‑β‑lactamase; NICC, Novel IC Card; OXA‑48, oxacilinase‑48; PCR, Polymerase Chain Reaction; VIM, Verona integron‑encoded metallo‑β‑lactamase.

**Table 3.** Comparison of PCR, AES and NICC in detecting single gene and multiple gene responsible for MBL resistance

Methods	Single gene detection	Multiple gene detection
**PCR**	19 isolates	81 isolates
**AES (Vitek-2)**	26 isolates	74 isolates
**O.K.N.V.I. Resist-5 card**	50 isolates	1 isolate

**Table 4.** Sensitivity and specificity of novel immunochromatography card versus PCR and AES

MBL	PCR positive	PCR negative	Sensitivity of novel IC card	Specificity of novel IC card	PPV*	NPV*
**OXA-48 and its variants** (95% CI: 48.8–70.5%)						
Novel IC card positive	47	5	58%	73.70%	90%	29.2%
Novel IC card negative	34	14	‒	‒	‒	‒
**NDM** (95% CI: 85.4–97.4%)						
Novel IC card positive	93	0	93%	100%	100%	0%
Novel IC card negative	7	0	‒	‒	‒	‒
**KPC** (95% CI: 61.5–99.8%)						
Novel IC card positive	7	4	87.50%	95.60%	64%	98.90%
Novel IC card negative	1	88	‒	‒	‒	‒
**VIM** (95% CI: 9.4–99.2%)						
Novel IC card positive	0	2	‒	98%	‒	99%
Novel IC card negative	1	97	‒	‒	‒	‒
**IMP** (95% CI: 0.8–90.6%)						
Novel IC card positive	0	1	‒	99%	‒	98%
Novel IC card negative	2	97	‒	‒	‒	‒

* PPV is the probability that a gene detected as *positive* by the novel IC card truly has the corresponding carbapenemase gene (i.e. it is a *true positive*). NPV is the probability that a gene detected as *negative* by the novel IC card truly does not have the corresponding carbapenemase gene (i.e. it is a *negative*).

## Discussion

CP organisms containing metallocene-lactamases including KPC-type, NDM, VIM, IMP or OXA have led to nosocomial or community-acquired infections in several countries [1,4]. NDM is a particularly prevalent *Enterobacterales* carbapenemase in India, subsequent to OXA-48-like, KPC, VIM and IMP [5,7]. OXA-48-type carbapenemases are the most common MBLs detected in non-fermenters like *A. baumannii* and *P. aeruginosa* [8,11]. Choosing suitable antimicrobial medicines for preventing infection requires rapid and precise identification of these five primary carbapenem resistance characteristics in clinical isolates [12]. While conventional PCR and sequencing, the gold standard method, can accurately identify and subtype carbapenemase genes, determining the carbapenemase type is sufficient for choosing suitable antimicrobial therapy. In CP organisms, Carbapenemase Inactivation and Carba NP exhibit notable concurrence rates with standard protocols (100% for *Enterobacterales* and 98.8% for non-fermenters) [13,15]. Phenotypic tests are undetermined and time-consuming due to incubation periods, while molecular procedures are expensive and require experienced staff, restricting their utilization. Given these drawbacks, this novel O.K.N.V.I RESIST-5 assay, which has been developed through multiplex IC lateral flow assays, has the following benefits: (i) a single, thorough test that could identify five clinically significant carbapenemases; (ii) no requirement for specialized equipment; (iii) simple and easy to interpret; (iv) rapid results.

This novel IC card assay has been evaluated in a retrospective study involving 164 CP MDR *Enterobacterales* at Belgium’s National Reference Laboratory. With sensitivities for NDM, VIM and IMP carbapenemases obtained at 91.2% (31 out of 34), 90% (36 out of 40) and 84.2% (16 out of 19), accordingly, in comparison to PCR, the assay effectively detected KPC and OXA-48-like carbapenemases [16]. In the present study, total MBL isolates which were tested with expanded gold standard protocol (PCR and AES) showed detection of 100% NDM (100 out of 100), 67% KPC (8 out of 12), 33% VIM (1 out of 3), 94% OXA-48 (81 out of 86) and 67% IMP (2 out of 3). In our investigation for accuracy of the novel IC card, we found that it identified 93% NDM (93 out of 100), 91.7% KPC (11 out of 12), 67% VIM (2 out of 3), 60% OXA-48 (52 out of 86) and 33% IMP (1 out of 3) carbapenemase producers. These data clearly state that detection of NDM, OXA-48 and IMP is better with PCR and AES compared to RESIST-5 novel IC card, while KPC and VIM MBL are more diagnosed with the RESIST-5 card compared to PCR alone, suggestive of false-positive detection by IC card. As per manufacturers' literature, it is mentioned that this novel IC card detects more than 15 variants of KPC, VIM, IMP and OXA, which increases its sensitivity and specificity [16]. However, the mentioned coverage of >15 variants per carbapenemase enzyme family is provided in manufacturer specifications, but they were not verified in the present experimental study. To highlight the importance of these variants, we need more multicentric studies to understand the coverage of these variants in different epidemiological settings and whether their appropriate detection with these rapid kits is possible or not. The assay’s challenge in identifying VIM and IMP originates from the encoding of these enzymes by gene cassettes embedded in class 1 or class 3 integrons. Integron cassettes are positioned within transposons, facilitating their insertion into bacterial chromosomes or plasmids; hence, the extracted antigen (purified protein) exhibits low expression during testing [17]. So, negative results need to be cautiously interpreted in cases where novel IC cards detected more OXA-48, KPC, VIM and IMP in *E. coli* compared to PCR and AES. However, PCR identified more NDM-producing isolates compared to the rapid card; this emphasizes that in India, the most common MBL is NDM, followed by OXA, so the chances of missing such common variants by novel IC are minimal, and it will not affect the clinical decision of treatment. The diagnostic performance of the new IC card kit for the detection of carbapenemase genes is target-dependent. The test displayed 100% PPV for NDM, showing full confidence in verifying that NDM was present when identified. In contrast, KPC detection had an exceedingly high sensitivity (87.5%) and specificity (95.6%), with an NPV (98.9%) that was conducive to ruling out KPC when negative. The PPV, however, was fair (63.6%), indicating that positive cases would require confirmatory testing. The performance of the IC card for OXA-48 was intermediate with fair sensitivity (58%) and specificity (73.7%) and good PPV (90.4%). Specificities (97.9 and 98.9%) and NPVs (98.9 and 97. 9%) were ‘high’ for VIM and IMP, respectively, and their PPVs were not interpreted appropriately due to lack of true positive VIM and IMP in the tested population. These findings suggest that the IC card seldom gives rise to false negatives for these genes, but all the positive detections were false, limiting its usefulness for verification of these infrequent resistance mechanisms.

Compared to prior research, a significant strength of the current investigation is its inclusion of a large number of CP isolates, encompassing over 100 clinical samples for various carbapenemase types with diverse genotypes and also comparing PCR results with the novel rapid card and AES of Vitek-2 system. Resistance to carbapenems, facilitated by carbapenemases, is conferred by genes that can be transmitted via plasmids or transposons. These genetic elements often carry additional resistance genes for quinolones and aminoglycosides, severely limiting safe treatment options [18]. The most prevalent and efficient carbapenemase genes globally are KPC, VIM, IMP, NDM and OXA-48 types [18,20]. Given this situation, we deem it necessary to review the current prevalence of carbapenemases, methods for their detection and the role of clinical microbiologists in managing and controlling these enzymes. However, a dispute remains since CLSI asserts that although molecular assays assist in identifying certain *β*-lactamase genes, they cannot exclude the existence of alternative *β*-lactamase genes, resistance mechanisms or novel variants with modified primer or probe binding sites. Therefore, CLSI recommends that phenotypic resistance be consistently documented. Moreover, CLSI indicates that existing clinical evidence is inadequate to ascertain the efficacy of carbapenem monotherapy against CP bacteria (identified via genotyping) with MIC within the susceptible range [20,21].

Ceftazidime-avibactam drug is most commonly used for isolates producing KPC and OXA type of MBL. Cefidericol or ceftazidime-avibactam aztreonam can be used for NDM-producing isolates. Without knowing the type of beta-lactamase isolates, we might initiate inappropriate treatment. This mistake can be avoided by the use of this novel IC card, which in the long term can prevent further resistance [21,22]. More so, the operational advantage, whether understanding the process of use of kit, requirement of training for implementation and visual identification of the carbapenemase band and time needed to perform, definitely gives this novel IC card the upper hand as a screening tool for diagnostic stewardship in critically ill patients. This immunochromatography card-based method does not require any sophisticated room or instruments like an incubator or molecular machines to perform the test.

## Limitations

During this study, we acknowledged the discrepant results among the isolates in which one or another method did not detect VIM, IMP or OXA MBL isolates, and that they should have been further tested using sequencing methods to resolve this result. However, due to budgetary constraints of this resource-limited, investigator-initiated study, we were unable to perform sequencing. Further studies are required for isolates producing VIM and IMP using this novel IC method and to check whether all variants as mentioned by the manufacturer’s literature are actually detected or missed. Considering PCR and AES as the expanded gold standard in this present study, we expect the VIM and IMP missed by PCR were actually false positives by the novel IC card. Limit of detection and MIC testing were not within the scope of this present study, because this study was focused on the evaluation of rapid resistance detection tools.

## Conclusion

The novel IC card assay offers a cost-effective, rapid, accurate and user-friendly approach to clinical microbiology practice. We believe that this novel IC card could be particularly beneficial in low-income countries and regions where NDM, OXA-48 and KPC carbapenemases are prevalent. It should be prospectively evaluated as a primary screening or rapid point-of-care test during a polyclonal nosocomial carbapenem resistant enterobacterales outbreak. We emphasized the need for future studies involving sequencing to validate these findings before using this novel card as a confirmatory tool.

## Abbreviations

AES: Advanced Expert System
CP: carbapenemase-producing
GNB: Gram-negative bacilli
IC: immunochromatographic
MBL: metallo-beta-lactamase
MDR: multi drug resistant
NPs: nanoparticles
NPV: negative predictive value
PPV: positive predictive value
TAT: turnaround time
